# Thoracic Discitis in Ankylosing Spondylitis

**DOI:** 10.7759/cureus.2972

**Published:** 2018-07-12

**Authors:** Eamon Maloney, Sachin Srinivasan, Timothy Shaver

**Affiliations:** 1 Internal Medicine/Pediatrics, University of Kansas School of Medicine - Wichita, Wichita, USA; 2 Internal Medicine, University of Kansas School of Medicine - Wichita, Wichita, USA; 3 Rheumatology, University of Kansas School of Medicine - Wichita, Wichita, USA

**Keywords:** ankylosing spondylitis, thoracic discitis, cauda equina syndrome

## Abstract

Chronic ankylosing spondylitis can lead to several rare long-term complications including cauda equina syndrome and inflammatory discitis especially without treatment. These complications are uncommon, but there is evidence that they can be treated with anti-tumor necrosis factor (TNF) inhibitors.

We present a case of a 52-year-old male with a 30-year history of undiagnosed ankylosing spondylitis with cauda equina syndrome on initial outpatient presentation with a negative lumbosacral magnetic resonance imaging (MRI). He was admitted later that month and was found to have thoracic discitis from MRI requiring emergent decompressive laminectomy. The neurosurgeon collected a culture of the surgical site which showed rare Gram-positive cocci on Gram stain. Infectious disease was consulted, and he was started on empiric vancomycin. The culture from the surgical site did not grow any organisms. Interventional radiology (IR) aspirated the T7-T8 disk area one week later. The initial Gram stain showed rare Gram-negative rods this time, and cefepime was added to the patient's antibiotic regimen. The culture from the disk aspiration again grew no organisms. Rheumatology was then consulted and hypothesized that the patient's discitis could be secondary to inflammation from long-standing ankylosing spondylitis. The hospitalist, infectious disease specialist, and rheumatologist reviewed the case and recommended a six-week course of vancomycin and cefepime despite the negative cultures as an infectious etiology could not be excluded. He did show some clinical improvement after surgery and was started on adalimumab following completion of empiric antibiotics.

This case highlights the difficulty in distinguishing between an infectious and inflammatory etiology for discitis in the setting of long-standing ankylosing spondylitis. The initiation of biological therapy without completely excluding the possibility of infection could lead to devastating consequences. It will likely be necessary to empirically treat for infection with these cases for the foreseeable future until there are more definitive tests to diagnose or exclude infectious discitis.

## Introduction

Ankylosing spondylitis has a few rare neurological complications, which include cauda equina syndrome and inflammatory discitis. Aseptic discitis in some cases can be the initial presentation of ankylosing spondylitis [1]. The treatment of these conditions is unclear as there are no randomized controlled trials or observational studies dedicated to addressing this question. One case report did show clinical and radiological improvement in inflammatory discitis with the use of anti-tumor necrosis factor (TNF) therapy [2]. Another report demonstrated effective treatment of cauda equina syndrome in ankylosing spondylitis with adalimumab [3].

The evaluation and treatment of discitis in ankylosing spondylitis can be further complicated by the potentially multifactorial etiology of discitis itself which can include infection, inflammation, or trauma. Inflammatory lesions tend to occur earlier in the disease process with involvement of multiple vertebral bodies, while traumatic lesions tend to occur later in the disease process with involvement of only a single vertebral level [4]. It can be very difficult, however, to distinguish discitis caused by infection and discitis caused by inflammation in long-standing ankylosing spondylitis. In this case study, we present a patient with ankylosing spondylitis who demonstrates this diagnostic conundrum.

## Case presentation

A 52-year-old male with a 30-year history of chronic low back pain of unknown etiology presented to a rheumatology clinic. He noted low back pain associated with morning stiffness that worsened throughout the day. He also described increased difficulty with ambulation associated with bilateral paresthesia of the lower extremities, saddle paresthesia, and increased urinary urgency. His past medical history was significant for hypertension, hyperlipidemia, and tonsillectomy as a child. He had no known history of trauma or fractures. He smoked for more than 30 years but denied any alcohol or drug use. His physical examination revealed severely limited range of motion of the cervical and lumbar spine with a modified Schober’s test of 2.4 cm. He also had significant weakness in his hip flexors with decreased patellar reflexes. He was positive for human leukocyte antigen (HLA) B27. Radiographs of the lumbar spine showed sacroiliitis and syndesmophytes in the lumbar spine. He was diagnosed with ankylosing spondylitis based on these results. He was also clinically diagnosed with cauda equina syndrome though a lumbar spine magnetic resonance imaging (MRI) done later did not show any cord compression. The recommendation was to start treatment with adalimumab pending the results of tuberculosis screening.

**Figure 1:**
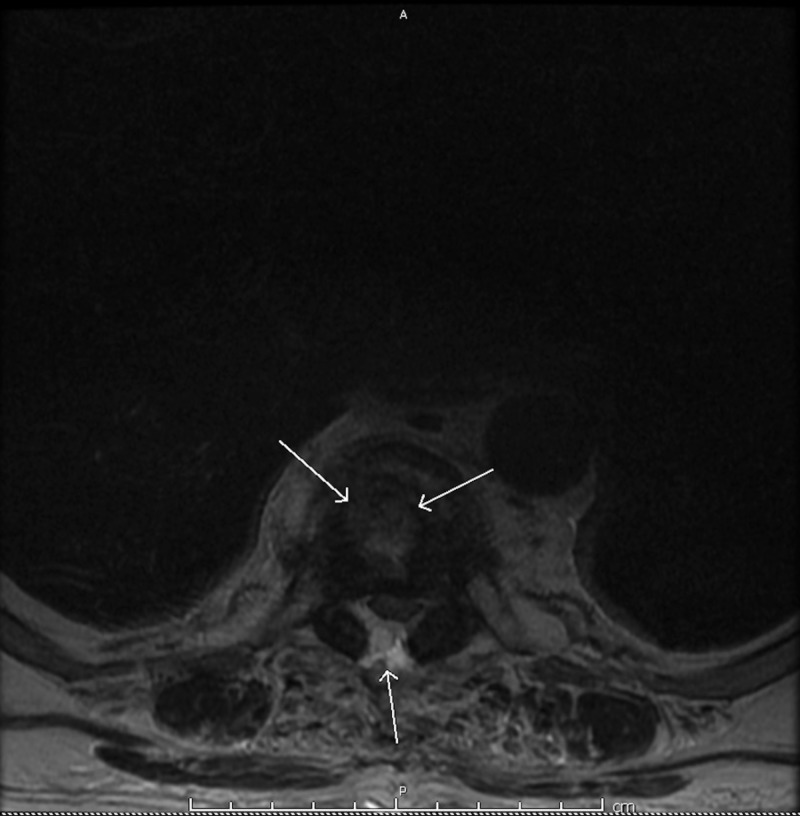
Transverse view of thoracic spine magnetic resonance imaging (MRI). MRI of thoracic spine with T7 discitis. Anterior arrows indicate inflammatory discitis while posterior arrow highlights spinal cord compression.

**Figure 2:**
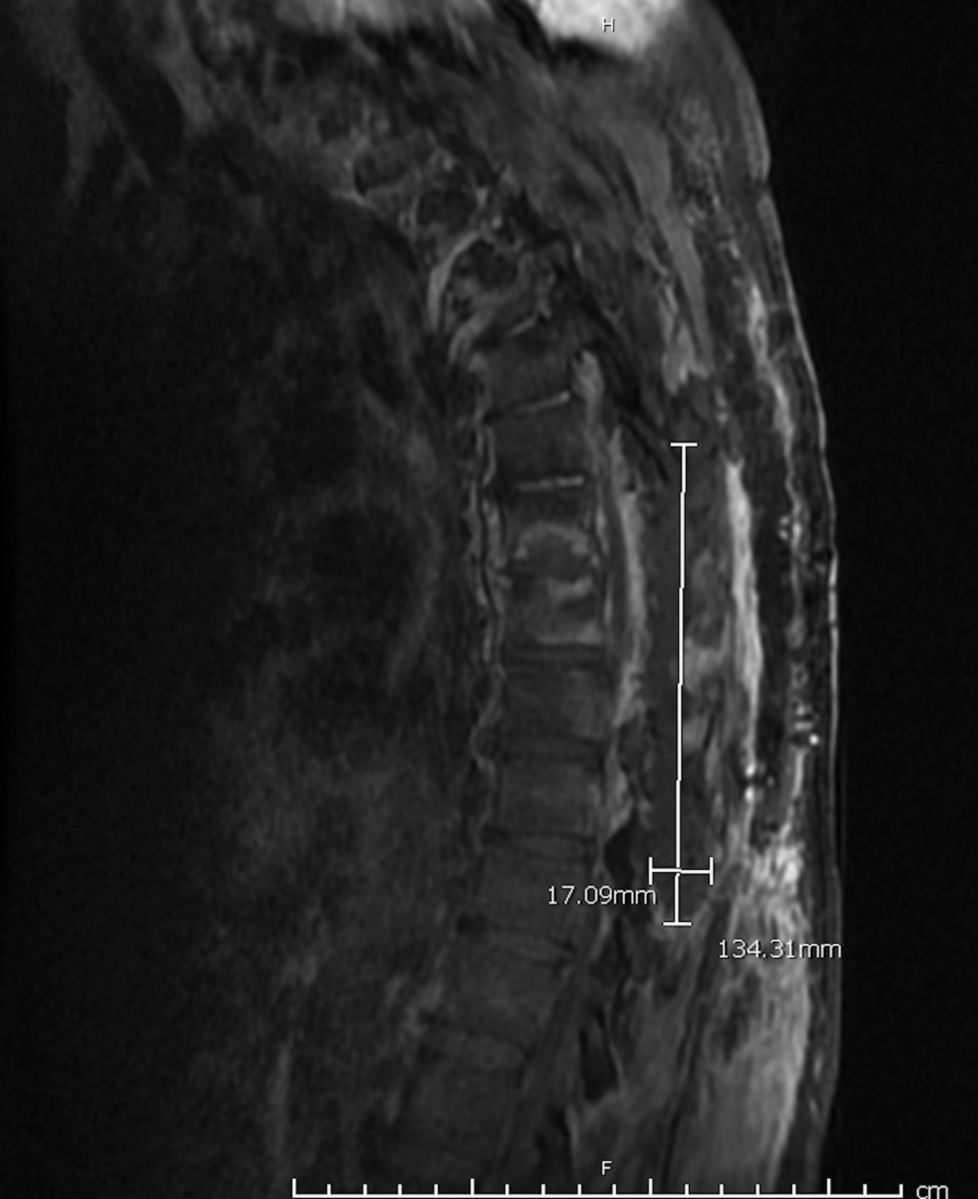
Sagittal view of thoracic spine magnetic resonance imaging (MRI). MRI of thoracic spine showing epidural phlegmon measuring approximately 13.4 cm by 1.7 cm.

While awaiting initiation of therapy, the patient had worsening lower extremity weakness and was admitted three weeks after presentation to the rheumatology clinic. He was afebrile on admission. His initial physical exam showed bilateral lower extremity weakness that was worse in the right lower extremity with bilateral diminished reflexes. His initial labs showed an elevated C-reactive protein (CRP) of 48 mg/L and no leukocytosis. A repeat lumbar spine MRI in the hospital showed no evidence of cord compression. His thoracic spine MRI, however, demonstrated T7-T8 discitis with concern for osteomyelitis but no pathological fracture. He underwent emergent surgical decompression of the thoracic spine from T7 to T10 (Figures 1-2). A swab of the surgical site showed rare Gram-positive cocci on Gram stain, and he was started on empiric vancomycin by the infectious disease specialist. His condition showed little improvement over the next few days, and the culture from his surgical specimen showed no growth. He then had interventional radiology (IR) aspiration of the T7-T8 disc space one week later that revealed rare Gram-negative rods on Gram stain. The infectious disease team started him on cefepime at that point and continued with vancomycin. The culture of the aspirated fluid again showed no growth on culture. Rheumatology was consulted and suggested that the etiology of the discitis could be inflammatory due to long-standing ankylosing spondylitis. This was supported by repeat lab evaluation two days after the IR aspiration which showed an increasing CRP of 129 mg/L despite antibiotic therapy as well as a low procalcitonin of 0.12 ng/mL. The hospitalist, infectious disease, and rheumatology teams all reviewed the case. The negative cultures and history of long-standing ankylosing spondylitis suggested sterile inflammatory discitis, but they could not definitively exclude an infectious etiology. The group then decided to complete a six-week course of vancomycin and cefepime prior to starting adalimumab due to the risks of starting immunosuppressive therapy in a patient with possible infectious discitis.

The patient was followed by infectious disease as an outpatient shortly after completing his six-week course of antibiotics. He showed a significant improvement in his lower extremity weakness and paresthesia, and his CRP had decreased to 35.6 mg/L. The infectious disease specialist then cleared him to start biological therapy. He started a course of adalimumab 40 mg subcutaneous every two weeks at his rheumatologist's office where he continued to show gradual improvement in both his ambulation and paresthesia. He will continue to be monitored over the next few months by his rheumatologist as he continues adalimumab therapy with repeat thoracic and lumbar spine MRIs in two months.

## Discussion

This case demonstrates the diagnostic dilemma that can occur with discitis in patients with long-standing ankylosing spondylitis. This patient had MRI findings concerning an infectious discitis, but his lack of fever, increasing inflammation despite antibiotic therapy, negative cultures, and negative procalcitonin were more suggestive of an inflammatory discitis. The utility of procalcitonin in infectious discitis requires more research, but one recent study did suggest it was less sensitive than CRP [5]. Our judgment was that it would be harmful to start immunosuppressive therapy in a patient if an infectious etiology cannot be completely excluded. Our patient subsequently did show clinical improvement after completing antibiotic therapy, but his surgical decompression may have played a significant role in his recovery.

Providers need to closely evaluate the patients having long-standing ankylosing spondylitis with discitis and be aware that it could be secondary to an infectious, inflammatory, or mixed etiology. The future of medicine will hopefully provide more specific tests or biomarkers to better distinguish between infectious and inflammatory discitis. Until that time, however, it remains prudent to hold off on treatment with immunosuppressive anti-TNF therapy and treat with antibiotics if an infectious etiology cannot be excluded from the differential.

## Conclusions

Inflammatory discitis and cauda equina syndrome are uncommon complications of ankylosing spondylitis that can be treated with adalimumab and other anti-TNF medications. It is difficult in some cases to distinguish between infectious and inflammatory discitis, and it remains necessary to treat with empiric antibiotics prior to starting anti-TNF therapy when an infectious etiology cannot be excluded.
